# Supplementary material for: Unravelling Metabolic Heterogeneity of Chinese Baijiu Fermentation in Age-Gradient Vessels
Source: Foods. 2023 Sep 14;12(18):3425. doi: 10.3390/foods12183425 (PMC10530105; doi:10.3390/foods12183425)

Tables

Table S1. The content of metabolites in three different *Digang*s.

	**Compounds**	**Contents（mg/kg）**																	
		A00	A05	A10	A15	A20	A25	B00	B05	B10	B15	B20	B25	C00	C05	C10	C15	C20	C25
1	Ethanol	25.09	698.39	1022.25	1390.02	1599.48	1597.59	26.73	787.73	1052.35	1312.06	1431.89	1529.28	25.09	754.92	1230.37	1393.89	1458.85	1835.31
2	Phenethyl alcohol	75.96	76.99	60.29	63.69	69.20	91.63	76.18	71.41	89.27	98.53	102.49	88.99	75.96	76.99	60.29	70.86	81.03	69.20
3	3-Methyl-1-butanol	43.01	26.13	53.72	56.24	50.24	51.75	11.45	67.72	73.85	72.97	75.45	68.63	43.01	28.47	54.61	54.28	54.02	50.24
4	2-Methyl-1-propanol	36.33	30.02	28.72	12.30	23.78	30.25	50.75	36.20	27.18	28.49	20.41	21.37	36.33	45.48	26.50	27.26	17.53	24.20
5	2,3-Butanediol	4.24	14.28	13.30	24.03	9.76	16.35	24.38	14.37	11.86	13.10	12.48	14.98	4.24	22.63	12.63	11.70	7.68	9.78
6	2-Furanylmethanol	4.26	19.77	12.37	13.98	0.00	0.00	30.62	12.24	11.47	0.00	0.00	0.00	4.26	21.96	13.58	1.18	0.00	0.00
7	1,2-Propanediol	23.17	2.45	2.25	2.00	1.46	2.59	0.00	1.67	1.27	1.54	1.74	0.00	23.17	3.13	1.84	1.44	1.51	1.95
8	3-(Methylsulfanyl)-1-propanol	10.14	2.64	1.46	1.11	0.06	1.28	2.31	1.56	1.32	1.32	1.34	1.28	10.14	2.58	1.32	13.28	1.29	0.84
9	Benzyl alcohol	3.50	0.00	0.00	0.00	0.00	0.00	2.67	0.00	0.00	0.00	0.00	0.00	3.50	0.00	0.00	0.00	0.00	0.00
10	Tetraethylene glycol	1.04	0.00	0.00	0.00	0.00	0.00	0.00	0.00	0.00	0.00	0.00	0.00	1.04	0.00	0.00	0.00	0.00	0.00
11	DL-sec-iso-Amyl alcohol	0.00	0.00	0.00	0.00	0.65	0.00	0.00	0.00	0.00	0.00	0.67	0.46	0.00	0.00	0.00	0.00	0.00	0.00
12	Ethyl oleate	9.40	1.66	4.70	5.57	6.55	5.70	6.21	2.67	4.17	5.12	6.13	5.24	9.40	7.25	3.91	5.28	6.35	5.57
13	2-Phenethyl isobutyrate	0.00	1.54	1.42	0.45	1.63	2.78	0.00	0.98	1.17	1.43	1.37	1.43	0.00	1.52	1.51	1.48	1.51	1.50
14	3-Methyl-1-butyl acetate	13.92	9.23	9.13	9.53	6.02	9.51	12.42	9.19	8.64	9.41	5.67	9.23	13.92	7.62	6.22	9.99	7.68	4.26
15	Ethyl acetate	22.35	108.13	157.88	216.99	240.30	297.07	19.68	110.72	160.28	176.28	195.25	234.95	22.35	80.24	153.07	183.17	179.62	218.92

16	Phenethyl acetate	0.83	0.00	0.00	0.00	0.00	0.00	0.24	0.00	0.00	0.00	0.00	0.00	0.83	0.00	0.00	0.00	0.00	0.00
17	Ethyl linoleate	11.73	0.00	0.00	0.00	0.00	0.00	0.00	0.00	0.00	0.00	0.00	0.00	11.73	18.46	0.00	0.00	0.00	0.00
18	Ethyl caprylate	71.32	27.96	77.67	92.23	77.85	89.84	19.18	61.59	69.41	96.13	76.70	74.35	71.32	51.17	74.12	98.32	90.32	74.97
19	Ethyl valerate	0.00	7.88	7.73	6.98	2.95	2.48	4.30	22.54	33.42	28.82	22.64	6.27	0.00	5.89	3.87	5.33	3.53	1.53
20	Z-10-dodecenyl acetate	0.51	0.00	0.41	0.30	0.93	3.38	0.24	0.00	0.54	0.59	0.70	9.38	0.51	0.00	0.37	0.39	0.53	0.50
21	Ethyl palmitate	15.72	7.07	11.00	11.71	5.82	16.21	12.01	8.20	12.54	12.16	14.40	14.46	15.72	15.23	9.16	11.24	16.58	15.93
22	Ethyl laurate	1.68	0.00	1.74	1.16	1.69	3.41	0.00	2.22	3.68	4.29	5.00	4.14	1.68	3.37	2.03	2.13	3.62	3.37
23	Ethyl lactate	230.77	44.28	111.49	107.64	143.95	246.13	219.58	55.01	83.72	141.35	179.99	255.94	230.77	41.37	147.83	136.02	182.50	254.88
24	Ethyl nonanoate	0.00	0.00	5.59	0.00	0.31	4.28	0.00	0.00	0.00	0.00	0.00	0.00	0.00	0.00	6.04	5.21	7.68	9.25
25	Ethyl hexanoate	27.67	38.51	49.21	46.51	18.32	14.33	21.83	59.94	95.27	93.22	57.91	23.72	27.67	40.29	23.63	47.68	29.42	14.37
26	Ethyl caprate	2.76	6.05	13.85	14.19	16.88	26.54	48.54	14.59	17.98	54.64	56.11	19.18	2.76	9.30	9.84	14.53	26.70	20.08
27	Ethyl heptanoate	0.00	0.00	0.00	0.00	0.00	0.00	0.00	0.00	0.00	0.00	0.79	0.00	0.00	0.00	0.00	0.00	0.00	0.00
28	Ethyl butanoate	6.22	23.95	9.62	13.69	6.94	7.47	4.13	22.15	36.50	38.47	28.99	12.45	6.22	24.98	6.92	12.19	7.66	4.93
29	Ethyl butanoate	0.00	0.31	0.47	0.00	0.00	1.85	0.00	0.37	0.59	1.00	1.13	1.40	0.00	1.17	0.41	0.56	1.24	0.86
30	Diethyl succinate	9.42	36.05	108.70	109.51	132.73	139.66	3.44	146.20	181.15	199.43	254.92	288.29	9.42	74.32	103.18	115.45	137.31	130.75
31	Ethyl phenylacetate	1.95	0.00	0.49	0.66	0.70	1.44	0.00	0.00	1.07	0.00	1.68	0.00	1.95	0.00	0.00	0.76	1.22	0.00
32	Phenethyl phenylacetate	0.00	0.00	0.00	1.57	0.11	0.00	0.00	0.00	0.00	0.00	1.46	0.00	0.00	0.00	0.00	0.00	0.00	0.00
33	Ethyl 3-methylpentanoate	0.00	0.00	6.75	0.00	5.45	0.00	5.48	6.19	10.00	9.35	0.98	0.00	0.00	0.00	0.00	0.00	0.00	0.00
34	Ethyl 2-hydroxy-4-methylvalerate	0.00	0.00	0.00	0.00	0.90	0.00	0.00	0.00	12.03	16.58	30.88	0.00	0.00	0.00	0.00	0.00	0.00	0.00

35	Propyl 2-methylvalerate	0.00	0.00	0.00	0.00	0.00	0.00	0.00	2.07	0.00	0.00	0.00	0.00	0.00	0.00	0.00	0.00	0.00	0.00
36	Methyl 2-methylpentanoate	0.00	2.21	0.00	0.00	0.00	0.00	0.00	0.00	0.00	0.00	0.00	0.00	0.00	0.00	0.00	0.00	0.00	0.00
37	Stearic acid	0.00	0.00	0.00	0.00	0.00	0.00	0.00	0.00	0.00	0.00	0.00	0.00	0.00	0.72	0.00	0.00	0.00	0.00
38	Acetic acid	13.26	41.52	60.00	48.37	38.55	36.32	15.52	43.16	60.59	46.48	44.24	41.28	13.26	33.66	44.24	35.72	38.39	27.74
39	Octanoic acid	0.00	2.32	1.65	1.00	0.63	1.27	0.00	2.11	1.82	1.41	1.39	1.00	0.00	3.74	2.16	0.48	0.49	0.47
40	Nonanoic acid	0.00	0.71	0.00	0.00	0.00	0.00	0.00	0.00	0.00	0.00	0.00	0.00	0.00	0.00	0.00	0.00	0.00	0.00
41	Hexanoic acid	0.00	2.40	1.44	1.82	3.31	0.87	0.00	1.81	2.02	2.17	2.29	0.97	0.00	3.27	1.91	1.13	4.91	0.83
42	Butyric acid	0.00	1.45	0.00	0.00	0.00	0.00	0.00	2.20	2.49	2.08	2.14	0.00	0.00	1.19	0.00	0.00	0.00	0.00
43	2-Ethylheptanoic acid	0.00	0.00	0.26	0.00	0.00	0.00	2.65	0.20	0.37	0.94	0.00	0.00	0.00	0.00	0.19	0.35	1.22	0.00
44	2-Methoxyphenol	0.00	0.00	0.00	0.00	0.84	0.14	0.00	0.00	0.00	0.00	0.00	0.18	0.00	0.00	0.00	0.00	0.97	0.11
45	2-Ethylphenol	0.00	0.55	0.00	0.50	0.00	0.00	0.00	0.63	0.00	0.00	0.00	0.00	0.00	0.00	0.64	0.00	0.27	0.64
46	4-Methylphenol	0.11	0.00	0.00	0.00	0.00	0.00	0.59	0.00	0.00	0.00	0.00	0.00	0.11	0.00	0.00	0.00	0.00	0.00
47	4-Methylguaiacol	1.78	1.35	0.97	1.14	0.34	1.99	0.37	1.46	2.12	3.94	5.89	6.27	1.78	2.41	1.28	1.46	1.97	1.84
48	4-Ethylphenol	0.28	0.00	0.52	0.00	1.77	0.65	1.71	0.00	0.85	0.97	1.28	1.13	0.28	0.00	0.00	0.49	0.51	0.00
49	4-Ethyl-2-methoxyphenol	2.67	2.64	2.02	2.07	0.48	0.52	0.44	3.30	4.29	5.74	7.93	0.70	2.67	4.41	2.17	2.57	3.62	3.26
50	Phenol	0.28	0.50	0.36	0.32	3.26	0.33	3.87	0.54	0.51	0.46	0.49	0.40	0.28	0.63	0.45	0.35	0.32	0.31
51	4-Hydroxy-3-methoxystyrene	1.10	2.33	1.33	1.93	1.43	1.28	0.00	1.33	2.29	2.43	2.11	0.64	1.10	2.76	1.30	2.48	3.06	1.98

52	2-Methoxy-4-propyl-Phenol	0.00	0.00	0.00	0.00	0.00	0.00	0.55	0.00	0.00	0.00	0.00	0.00	0.00	0.00	0.00	0.00	0.00	0.00
53	2-Methoxyacetophenone	7.77	0.00	0.00	10.41	16.22	3.95	0.00	0.00	0.00	0.00	3.63	0.00	7.77	0.00	16.12	4.75	8.12	2.87
54	2-Amylfuran	0.00	2.70	1.25	0.00	0.00	0.00	3.50	0.00	0.00	0.00	0.00	0.00	0.00	3.35	0.00	0.00	0.00	0.00
55	3-Methyl-2(5H)-furanone	0.00	0.15	0.06	0.07	0.00	0.00	0.00	0.15	0.06	0.07	0.00	0.00	0.00	0.15	0.06	0.07	0.00	0.00
56	3-Hydroxy-2-oxobutane	0.00	3.27	11.69	0.00	0.00	0.00	0.00	2.73	5.48	0.00	0.00	0.00	0.00	6.08	15.77	1.23	0.00	0.00
57	Benzaldehyde	7.85	4.23	4.04	4.59	7.51	7.28	6.12	3.07	4.78	10.03	11.08	9.89	7.85	4.82	3.10	3.80	7.34	7.49
58	Phenylacetaldehyde	0.76	4.49	9.41	7.37	9.74	0.00	0.00	0.00	0.00	0.00	0.00	15.53	0.76	11.99	6.59	9.13	8.05	27.13
59	Mesyl anhydride	0.00	0.00	2.28	2.50	1.71	1.28	0.00	1.87	2.34	1.48	1.86	1.31	0.00	1.63	2.58	2.43	1.26	1.42
60	Oxime-, methoxy-phenyl-	0.00	1.61	1.61	1.91	1.77	2.23	0.98	3.69	2.85	2.80	1.83	1.74	0.00	1.74	1.55	2.41	1.36	0.92
61	(E)-β-Farnesene	0.00	1.05	0.69	0.75	0.76	0.00	0.00	0.00	0.90	1.02	0.00	0.00	0.00	1.37	0.62	0.74	0.92	0.75
62	Furfural	0.00	0.00	0.00	0.00	0.00	0.00	3.03	0.00	0.00	0.00	0.00	0.00	0.00	0.00	0.00	0.00	0.00	0.00
63	Nonanal	0.00	0.00	0.00	0.00	2.84	0.00	0.00	0.00	0.00	0.00	0.00	0.00	0.00	0.00	0.00	0.00	0.00	1.70
64	Tetramethylpyrazine	0.00	0.00	0.00	0.00	0.00	0.00	0.00	0.00	0.00	0.00	0.00	0.00	0.00	0.00	0.28	0.00	0.00	0.00

Table S2. Bacterial and fungal microbiota diversity index based on 16S rRNA and ITS amplicons sequencing across samples.

	ID	A05	A10	A15	A20	A25	B05	B10	B15	B20	B25	C05	C10	C15	C20	C25
Bacteria	shannon	2.830	2.157	2.333	0.464	0.725	2.104	0.099	0.070	0.008	0.004	2.583	2.156	0.170	0.188	0.951
	simpson	0.789	0.604	0.737	0.124	0.237	0.627	0.017	0.013	0.001	0.001	0.761	0.724	0.034	0.045	0.352
	chao1	45.667	65.143	50.429	24.200	12.500	74.000	19.250	8.250	4.000	2.000	31.000	32.670	13.600	6.000	9.500
	goods coverage	0.997	0.995	0.996	0.998	0.999	0.993	0.998	0.999	0.999	1.000	0.998	0.997	0.999	1.000	0.999
Fungi	shannon	1.747	0.975	0.964	0.600	0.123	0.575	0.408	0.200	0.336	0.250	2.196	0.443	0.346	0.255	0.400
	simpson	0.556	0.392	0.432	0.160	0.023	0.180	0.115	0.053	0.076	0.055	0.668	0.118	0.083	0.065	0.108
	chao1	28.200	13.500	17.500	14.500	28.000	28.000	13.500	7.000	30.000	21.000	35.200	23.250	36.000	14.000	10.250
	Goods coverage	0.999	1.000	0.999	0.999	0.998	0.999	0.999	1.000	0.998	0.999	0.999	0.999	0.998	0.999	1.000

Table S3. Comparison of prediction performance of different models.

Model	Ethyl lactate		Ethyl acetate	
	MAPE	R^2	MAPE	R^2
ANN	26.517	0.645	15.925	0.481
DT	19.121	0.508	25.051	0.289
SVR	33.395	0.604	19.477	0.453

Table S4. Predicted values of 19 differential metabolites.

Compounds	Contents (mg/kg)				
	5	10	15	20	25
Phenethyl alcohol	55.58	82.96	78.38	96.61	95.17
3-Methyl-1-butanol	47.21	66.17	66.25	73.21	70.05
2-Methyl-1-propanol	26.83	36.20	35.96	19.11	22.46
2,3-Butanediol	13.25	14.02	12.45	12.47	13.95
3-(Methylsulfanyl)-1-propanol	2.15	1.94	2.15	1.39	1.27
Ethyl oleate	3.64	0.80	0.29	5.36	5.68
Ethyl acetate	130.21	200.28	187.63	187.03	246.89
Ethyl caprylate	68.52	80.71	80.58	66.79	81.67
Ethyl valerate	6.76	34.15	34.36	25.92	4.67
Ethyl palmitate	6.77	11.98	11.00	13.57	13.96
Ethyl laurate	0.18	2.30	1.84	4.08	4.32
Ethyl lactate	121.37	126.62	102.57	164.85	239.08
Ethyl nonanoate	2.87	3.23	2.99	0.08	0.20
Ethyl hexanoate	41.24	83.11	83.83	56.91	28.42
Ethyl caprate	11.54	19.38	19.38	37.77	29.20
Ethyl butanoate	5.64	37.26	38.12	28.35	13.91
Diethyl succinate	92.38	78.63	75.20	234.80	275.01
4-Ethyl-2-methoxyphenol	2.45	2.31	2.63	5.17	4.18
3-Hydroxy-2-oxobutane	9.49	11.58	11.71	2.63	-1.33

Figure captions

Figure S1. Venn diagram for metabolites in three *Digang*s.

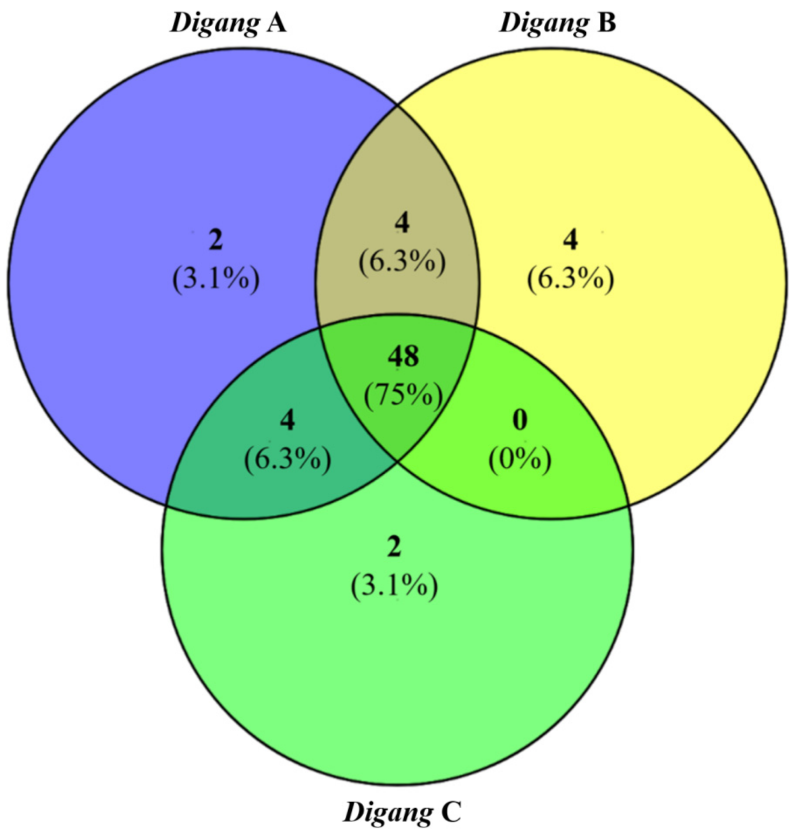

Figure S2. Comparison of aroma profiles of three *Digang*s. Significance: * $p < 0.05$.

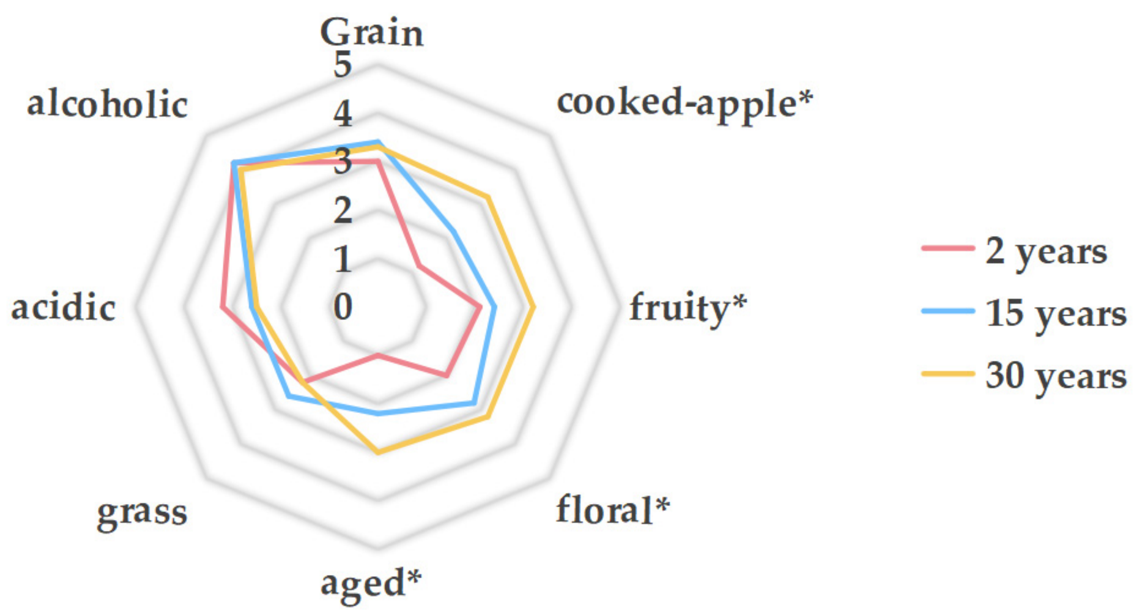

Figure S3. Venn diagram for microorganism genus in three *Digang*s. (a) Venn diagram for bacterial genera in three *Digang*s. (b) Venn diagram for fungal genera in three *Digang*s.

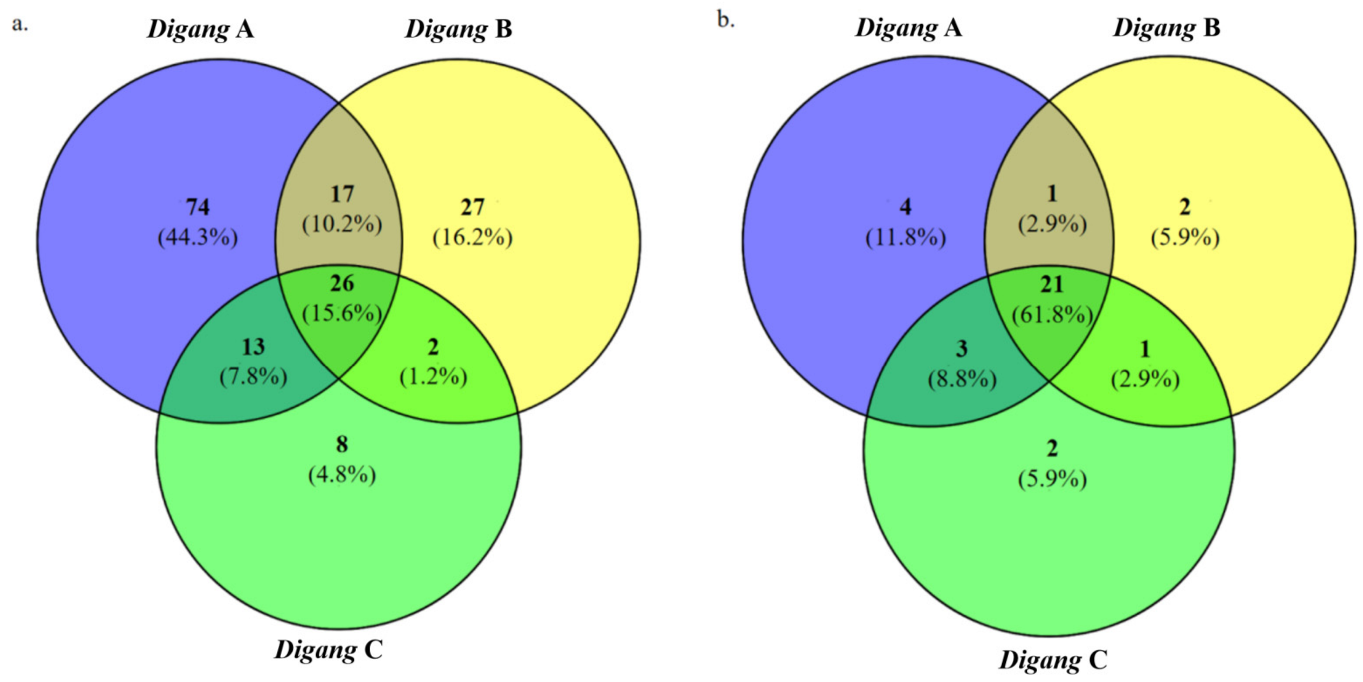

Figure S4. Fermentation temperature in three *Digang*s.

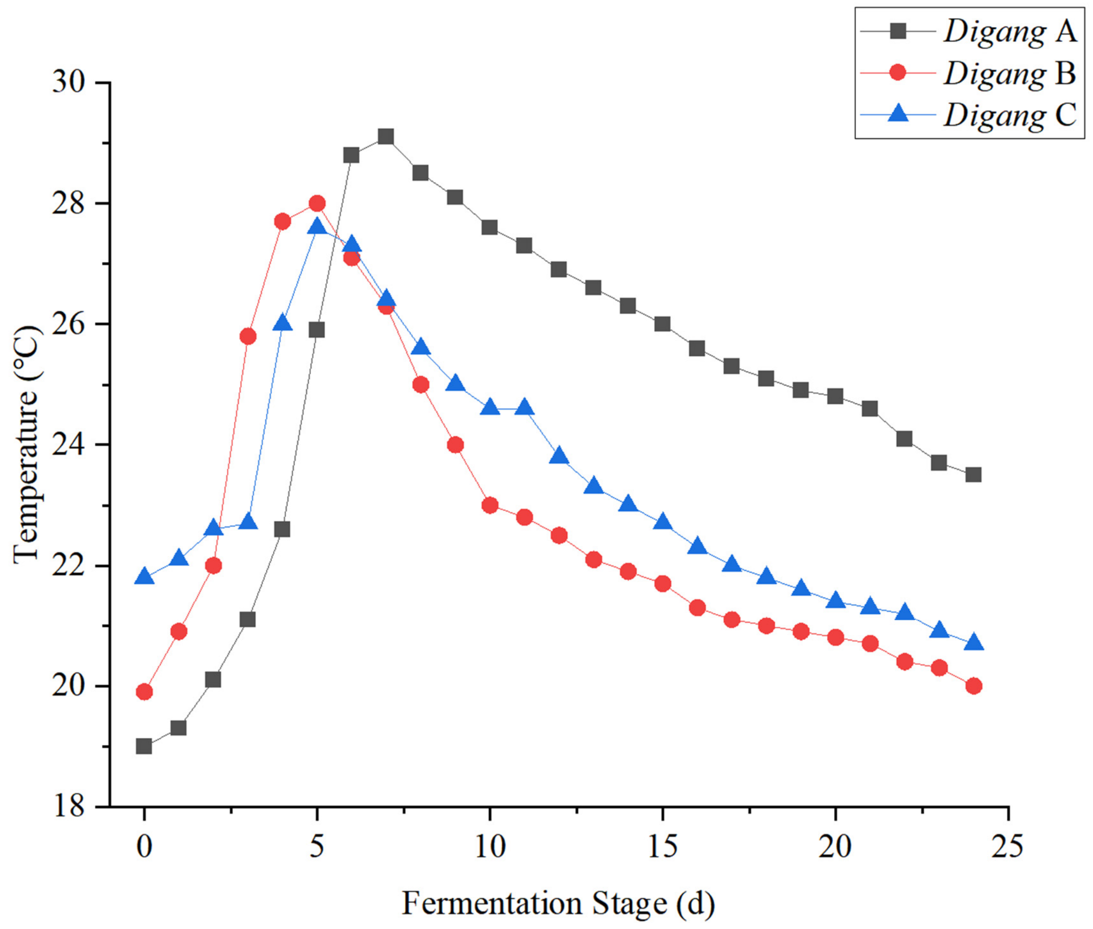

Supplement: Supplementary file 1 [file foods-12-03425-s001.zip › Supplementary Materials.pdf]
